# Notch Signaling Activation as a Hallmark for Triple-Negative Breast Cancer Subtype

**DOI:** 10.1155/2019/8707053

**Published:** 2019-07-11

**Authors:** M. V. Giuli, E. Giuliani, I. Screpanti, D. Bellavia, S. Checquolo

**Affiliations:** ^1^Department of Molecular Medicine, “Sapienza” University of Rome, Rome, Italy; ^2^Department of Medico-Surgical Sciences and Biotechnology, Sapienza University, Latina, Italy

## Abstract

Triple-negative breast cancer (TNBC) is a subgroup of 15%-20% of diagnosed breast cancer patients. It is generally considered to be the most difficult breast cancer subtype to deal with, due to the lack of estrogen receptor (ER), progesterone receptor (PR), and human epidermal growth factor receptor 2 (HER2), which usually direct targeted therapies. In this scenario, the current treatments of TNBC-affected patients rely on tumor excision and conventional chemotherapy. As a result, the prognosis is overall poor. Thus, the identification and characterization of targets for novel therapies are urgently required. The Notch signaling pathway has emerged to act in the pathogenesis and tumor progression of TNBCs. Firstly, Notch receptors are associated with the regulation of tumor-initiating cells (TICs) behavior, as well as with the aetiology of TNBCs. Secondly, there is a strong evidence that Notch pathway is a relevant player in mammary cancer stem cells maintenance and expansion. Finally, Notch receptors expression and activation strongly correlate with the aggressive clinicopathological and biological phenotypes of breast cancer (e.g., invasiveness and chemoresistance), which are relevant characteristics of TNBC subtype. The purpose of this up-to-date review is to provide a detailed overview of the specific role of all four Notch receptors (Notch1, Notch2, Notch3, and Notch4) in TNBCs, thus identifying the Notch signaling pathway deregulation/activation as a pathognomonic feature of this breast cancer subtype. Furthermore, this review will also discuss recent information associated with different therapeutic options related to the four Notch receptors, which may be useful to evaluate prognostic or predictive indicators as well as to develop new therapies aimed at improving the clinical outcome of TNBC patients.

## 1. Introduction

Breast cancer is the most commonly diagnosed cancer in women worldwide [1, 2]. The presence or absence of estrogen receptors (ERs), progesterone receptors (PRs), and the human epidermal growth factor receptor 2 (HER2/neu) classifies breast cancer in different subtypes [3]. Hormone receptor positive breast cancers represent 60% of all breast cancers [4], while the lack of expression of ER, PR, and HER2 characterizes TNBC subtype [5, 6], which accounts for 15-20% of breast cancer cases.

TNBCs predominantly affect younger patients (< 40 years) and are more frequent in African-American women, where they are associated with BRCA gene mutations [7, 8]. They are heterogeneous tumors with aggressive phenotype and higher relapse rate. Moreover, compared to other BC subtypes, TNBCs are less differentiated [8, 9] and prone to metastasize within 5 years of diagnosis [8]. Furthermore, TNBC-bearing patients have a shorter overall survival when compared to other BC subtypes [7, 10]. The intertumoral and intratumoral heterogeneity represent one of the major challenges for the efficacy of the treatment of this cancer. Lehmann and colleagues classified TNBC into six different subtypes by analyzing their gene expression profiles: the basal-like (BL1 and BL2), mesenchymal (M), mesenchymal stem-like (MSL), immunomodulatory (IM), and luminal androgen receptor (LAR)-enriched tumors [9]. Since TNBCs patients are characterized by this molecular heterogeneity, chemotherapy (anthracycline and taxane-based treatments also with platinum agents addiction) represents the primary systemic treatment. Moreover, although combination therapies have ameliorated the response rates, this improvement leads to increased toxicity and multidrug resistance. On the basis of the stratification of TNBCs into subtypes, many preclinical and clinical trials are allowing the development of new targeted therapies to treat the 60–70% of patients who do not respond to chemotherapy [11]. These alternative approaches include the use of PARP and tyrosine kinase receptor inhibitors, the targeting of Wnt/*β*-catenin or PI3K/AKT/mTOR pathway, the emerging immunotherapy, and the use of epigenetic drugs and androgen receptor (AR) antagonists [12], as described below in more detail.

In this scenario, since it has been demonstrated that Notch signaling plays an important role in breast cancer cell growth, migration, invasion, and metastasis, and its aberrant activation is associated with a poor prognosis, resistance to treatments, and relapse [13], here we discuss the therapeutic potential of targeting Notch signaling in breast cancer treatment, focusing on the TNBC field. Until now, a lot of effort has been made to find the optimal pharmacological Notch inhibition, as the typical approach to target Notch pathway is mainly based on *γ*-secretase inhibitors (GSIs) use [14], which however represents a pan-Notch inhibitor drug strongly associated with severe gastrointestinal toxicity [15]. Inhibition of a specific receptor alone may reduce or avoid toxicity, thus showing a clear advantage over pan-Notch inhibitors. Although the Notch signaling pathway has been widely studied, the specific role of the individual Notch receptor in cancer is still unclear.

**Figure 1:**
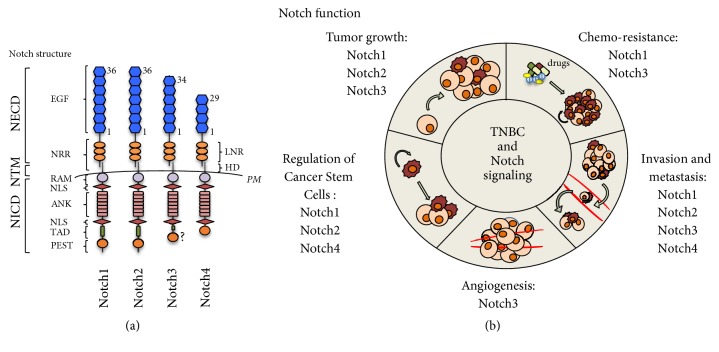
*Role of Notch signaling in TNBC.* (a) Schematic representation of the Notch receptors structure. Abbreviations. NECD: Notch extracellular domain; NTM: Notch transmembrane; NICD: Notch intracellular domain; EGF: epidermal growth factor-like repeats; NRR: negative regulatory region; LNR: Lin12/Notch repeats; HD: heterodimerization domain;* PM*: plasmatic membrane; RAM: RBP-j associated molecule; NLS: nuclear localization signal; ANK: ankyrin repeats; PEST: proline (P), glutamic acid (E), serine (S), and threonine (T). (b) The cartoon schematically depicts the involvement of each Notch receptor on TNBC initiation and progression.

In this review we summarize (and discuss) the current knowledge of the role of each individual Notch receptor in TNBC (Figure 1), in order to suggest the identification of drugs targeting specific Notch(s) with an effective anticancer potential and low toxicity, trying to direct future directions in this challenging field (Table 1).

## 2. Notch Signaling Overview in TNBC

### 2.1. Notch Structure and Function

Juxtacrine signaling is pivotal in several developmental processes and relies on communication between one cell and a neighboring cell through the interaction of transmembrane receptors and ligands [16]. The Notch signaling pathway is an example of this short-range cell-cell communication and plays an essential role in metazoan development [17]. The Notch receptor is a single-pass transmembrane protein expressed on the plasmatic membrane as a processed heterodimer after the cleavage by furin-like protein convertase in the Golgi compartment [18]. It was discovered in Drosophila melanogaster. The fly genome encodes only one Notch protein while two receptors, which have redundant roles, were identified in Caenorhabditis elegans [19]. In contrast, mammals have four Notch paralogs that only partly share the same functions [20] and this is due to their variable structural homology [21].

Regarding the structural organization of the Notch receptors (Figure 1(a)), they share a three-domain structure: an extracellular domain (NECD), a transmembrane region (NTM), and an intracellular domain (NICD) which translocates to the nucleus after two sequential proteolytic cleavages triggered by ADAM metalloproteases and a *γ*-secretase complex, respectively. According to the canonical Notch signaling model, these events are due to the interaction between the receptors and their ligands [21], expressed on neighboring cells.

The NECD contains 29 to 36 epidermal growth factor-like (EGF-like) repeats which are responsible for the ligands binding [22], the negative regulatory region (NRR), consisting of three cysteine-rich LNR Notch repeats, and the hetero-dimerization domain (HD), which prevents receptor activation in the absence of ligands [23]. The NTM region contains a *γ*-secretase cleavage site which is critical for signal activation [24]. The NICD consists of a RAM (RBP-j-Associated Molecule) domain, ankyrin (ANK) repeats flanked by two nuclear localization signals (NLS), a transcriptional activation domain (TAD), and a C-terminal Pro-Glu-Ser-Thr (PEST) domain which is the substrate of ubiquitin ligases that target the NICD for proteasomal degradation [25]. Both RAM ad ANK domains are necessary to recruit transcriptional coactivators within the nucleus [26] (Figure 1(a)).

In mammals, the five Notch ligands, Dll1, Dll3, and Dll4 (members of the Delta family of ligands) and Jagged1 and Jagged2 (members of the Serrate family of ligands), are single-pass transmembrane proteins [27]. Dll3 gene encodes a decoy receptor and, as a result, it is not able to activate Notch receptors in-trans [28].

Notch signaling has pleiotropic effects during development and in adult tissues, in spite of the simplicity of the core pathway [29]. As a matter of fact, Notch activity affects both proliferation and cell death and drives differentiation and acquisition of specific cell fates. Furthermore, it is involved in the maintenance of stem cells [30].

Since the Notch receptors is central for these processes, its deregulation has been implicated in the development of congenital diseases [31] or cancer, as either oncogenes or tumor suppressors [32, 33].

Specifically, Notch signaling pathway is involved in mammary development and homeostasis as well as in the promotion of breast cancer when dysregulated [34]. Indeed, accumulating evidence sustains the importance of Notch pathway in mammary stem cells (MaSCs) generation and maintenance during mammary gland development [35]. This process normally takes place over a period of rapid growth during puberty and, subsequently, it undergoes cycles of expansion and regression with each estrous cycle, pregnancy, lactation, and involution until menopause [36]. In this scenario, it has been demonstrated that Notch pathway plays a fundamental role in regulating both self-renewal [37] and differentiation of MaSCs [38, 39], thus allowing mammary gland homeostasis. Thus, the aberrant activation of Notch signaling has been shown to be an early event in breast cancer development [37]. A TCGA breast cancer data was analyzed for mutations in Notch receptors genes [40]. Among the 956 breast tumor samples analyzed, there were 42 mutations in Notch genes: 25 of them are clustered in the HD or lead to a PEST domain disruption, finally leading to NICD overexpression [40, 41]. In addition, compared to normal tissues, a lower expression of known Notch negative regulators in breast cancer was frequently found [42, 43]. In particular, FBXW7 mutations were significantly more frequent in TNBC compared to other breast tumor subtypes [44] and these determine an increased NICD stability, thus correlating with poorer prognosis of breast cancer-bearing patients [45]. Moreover, a novel molecular mechanism that correlates low NUMB expression with high Notch activity in the regulation of breast tumor EMT, especially in TNBCs, was found [46].

In keeping with these findings, the role of Notch signaling in breast cancer initiation and progression has been extensively studied and most of the reported data highlights its oncogenic role in breast cancer [47].

### 2.2. Role of Notch Paralogs in TNBC

#### 2.2.1. Notch1

The first demonstration that Notch receptors are oncogenes also in regard to breast cancer derives from studies on murine models. In particular, the Notch1 gene was identified as a novel target for mouse mammary tumor virus (MMTV) insertional activation, thus leading to the overexpression of Notch1 mutated forms, finally involved in mammary tumor formation [48]. Compared with normal tissues, Notch1 is fairly expressed in human breast cancer and its elevated expression represents an early event during carcinogenesis, as it has been demonstrated that the enforced expression of ectopic N1ICD contributes to the incidence and development of breast cancer [49], being predictive of poorest overall patient survival [50–52]. Several studies have related Notch1 signaling to TNBCs [53]. In particular, the basal-like 1 (BL1) and mesenchymal stem-like (MSL) subtypes are characterized by the high expression of this receptor [54, 55], strongly correlated with ominous outcomes of tumor [56].

Notch pathway is aberrantly activated via multiple mutational mechanisms and it is liable of TNBC tumor development. Although PEST domain mutations in Notch1 protein mainly regard oncogenic events in T-ALL [57], around 13% of TNBC exhibits in-frame deletions of Notch1 exons 21-27, which disrupt the NRR and HD domains, thus leading to upregulation of its pathway caused by either ligand-independent receptor activation or N1ICD half-life extension [40]. As a consequence, Notch1-mutated-TNBCs show a strong overexpression of Notch1 target genes, like NOTCH3, HES1, HEY2, MYC, CCND1, HES4, NRARP, and NOTCH1 itself, in comparison with Notch1 wild-type tumors, thus resulting in oncogenic phenotype of TNBCs [40]. In addition, a correlation has been found between the expression of Notch1 protein and known prognostic factors in breast cancer, analyzed by IHC assay in 115 breast cancer tissues [58]. The presence of Notch1 in tumor tissue was significantly associated with TNBC subtype (P=0.041), high metastasis rate (P=0.035), tumor-node-metastasis (TNM) stages, and ALDH1* status*, a known marker of cancer stem cells (CSCs).

Furthermore, a significant positive correlation was observed between Notch1 protein and both AKT and NF-*κ*B proteins activation in preclinical models, thus finally promoting TNBC cell growth, migration, and invasion [59]. Interestingly, more recently Hossain and colleagues described in detail noncanonical mechanisms downstream of Jagged-1-mediated Notch1 activation that trigger AKT phosphorylation, NF-*κ*B activation, and mitochondrial metabolism, thus leading to the transcription of survival genes in TNBC cells [60]. In agreement with these data, it has been demonstrated that* Genistein*, a phytochemical originally isolated from soybean, by inhibiting Notch1, affected MDA-MB-231 TNBC growth through modulating NF-*κ*B activity [61].

It is well demonstrated that CSCs are involved in initiation, progression, and chemotherapy resistance of cancers [62, 63].

Notch1 appears to be in part responsible for maintaining CSC stemness in TNBCs, and the specific inhibition of its signaling has a remarkable inhibitory effect on this cancer subtype, thus increasing the sensitivity of TNBC to chemotherapeutic reagents [64]. It is reported that in HCC70, SUM149, and MDA-MB-231 TNBC cell lines, the c-Jun N-terminal kinase (JNK) protein promotes CSC self-renewal and maintenance via transcription of Notch1, whose activation affects migration and invasion of tumor cells [65]. In accordance with these findings, both JNK and Notch1 knockdown significantly reduced mammosphere formation in TNBC cells [65]. Moreover, Mittal and colleagues, by using a novel monoclonal antibody to inhibit Notch1 (MAb602.101), observed a significant reduction in tumor growth and in the number and sizes of mammospheres compared to controls, thus resulting in the depletion of the putative cancer stem-like cell subpopulation [66]. Furthermore, Bhola and colleagues demonstrated that resistance to TORC1/2 inhibition in TNBC is driven by Notch1 activation whose expression is increased in response to treatment. In consequence, genetic and pharmacological blockade of Notch1 is able to revert the increase in CSC markers expression, mammosphere formation, and tumor-initiating ability, all induced during TORC1/2 inhibition treatment [67]. All these studies sustained an important correlation between Notch1 inhibition and the restoration of the sensitivity to drug treatments, thus showing interesting findings which would improve the efficacy of conventional therapies by directly targeting the CSC niche [64, 68]. In addition, significant upregulated Notch-1 protein levels are found in Doxorubicin resistant MCF-7 cells compared to parental sensitive MCF-7 cells [69]. In keeping with these data, Notch1 inhibition enhanced the antitumor effects of Paclitaxel, the first-line chemotherapeutic drug for clinical treatment of TNBC, in both MDA-MB-231 and MCF-7 chemoresistant cells [70].

Emerging evidence demonstrated the involvement of Notch1 also in the invasion and migration steps which characterize the epithelial-to-mesenchymal transition (EMT) process in TNBC [71]. The authors observed that Notch1 is negatively regulated by miR-3178, which is significantly lower in TNBCs when compared to the other subtypes: the lower levels of miR-3178 lead to increased Notch1 activity followed by increased Snail1 expression, which finally contributes to EMT regulation [71]. Indeed, the inhibition of Slug/Notch1 signaling axis, by regulating EMT process, seems to be sufficient to decrease tumor-initiating cells (TICs) number, tumor induction, and metastasis [72]. In keeping with these data, Notch1 expression is higher in Cisplatin-resistant MDA-MB-231 TNBC cells, compared to the parental cells, and this helped to induce chemoresistance via activating AKT pathway and promoting EMT [73]. Furthermore, it has been demonstrated that the combined treatment with Doxorubicin plus GSIs of the same resistant cells, besides downregulating Notch-1, is also able to decrease both Cyclin D1 and antiapoptotic protein Bcl-2 while upregulating PTEN and proapoptotic proteins, finally leading to synergistic antitumor effects* in vitro* and* in vivo* TNBC xenografts models [74].

More recently, Lee and colleagues demonstrated that Notch1 inactivation, obtained as a consequence of the knockdown of Tribbles Homolog 3 (TRIB3) protein in MDA-MB-231 and AS-B244 radio-resistant TNBC cells, correlated with a cell resensitization toward radiation therapy [75]. Interestingly, some studies showed a Notch1 involvement in metabolic alterations of cancer cells. Abnormal mitochondrial fission is implicated in the development and progression of many human cancers [76] and Notch signaling has been reported to be closely related to mitochondrial network and function in different cellular contexts [77–79]. Dynamin related protein (Drp1) is involved in mitochondrial fission while Mitofusin-1 (Mfn1) is a mitochondrial membrane protein that participates in mitochondrial fusion, thus contributing to the maintenance of the mitochondrial network. Perumalsamy and colleagues identified the N1ICD-Akt-Mfn signaling cascade as a novel pathway regulating cell survival, in a way independent of the canonical functions associated with N1ICD activity, thus demonstrating the Notch1 involvement in mitochondrial network and apoptotic resistance in HeLa cells [79]. More recently, it was demonstrated that the Notch1/Mfn2 pathway was able to favor the protective effect of melatonin on myocardial infarction, by using both* in vitro* and* in vivo* models [78]. In TNBC context, Chen and colleagues demonstrated that the observed increase in the mitochondrial fission, characterized by the combined upregulation of Drp1 and downregulation of Mfn1, was due to a positive feedback loop closely dependent on Notch1 protein: mitochondrial fission contributes to activation of Notch1, which in turn promotes and amplifies the mitochondrial fission through the maintenance of both Drp1 and Mfn1 altered expression. This process strongly correlated with TNBC progression and a poorer overall survival of TNBC-bearing patients [80].

All these studies suggest that activation of the Notch1 pathway is a key event in TNBC etiology and it contributes to the development and progression of malignant phenotype of TNBC subtype.

#### 2.2.2. Notch2

The role of Notch2 in breast cancer is less well characterized with respect to Notch1. Previous studies reported that Notch2 increases tumorigenicity in thymic lymphoma [81] and in embryonal brain tumor cell lines [82]. Conversely, Notch2 signaling causes cells growth arrest in small cell lung cancer [83]. Therefore, the cellular context is important for tumorigenic outcome of Notch2 signaling. Notch2 can play a different role in TNBCs, thus acting as an oncogene or tumor suppressor. Evidence for its oncogenic role came from studies on cultured breast cancer cells where knockdown of Notch2 leads to the inhibition of cell migration and cancer stem cell survival [84, 85]. In particular, Kim and colleagues revealed that treatment of MCF-7, MDA-MB-231, and SUM159 human breast cancer cells with Benzyl isothiocyanate (BITC), a constituent of cruciferous vegetables, increases levels of the active form of Notch1, Notch2, and Notch4 in both cultured and xenografted cells. In this scenario, only Notch2 activation is able to impede inhibitory effect of treatment on cell migration [85]. In keeping with these data, the proapoptotic effect of Zerumbone (ZER), a sesquiterpene isolated from subtropical ginger, on TNBC cells was counteracted by Notch2 activation and significantly increased upon its knockdown [86].

Analysis of Notch2 expression in normal mammary tissue and breast tumors, in association with clinical data, also sustained a tumor suppressor function for this receptor. The most convincing evidence for this Notch2 capability is provided by O'Neill and colleagues [87]. They reported that overexpression of N2ICD in MDA-MB-231 cells is potently able to suppress tumor growth both* in vitro* and* in vivo* in xenografts. Therefore, Notch2 plays a role in the inhibition of mammary adenocarcinoma growth, mostly in comparison with Notch4 ICD in the same context. Another study revealed that the* in vivo* growth of MDA-MB-231 and SUM159 xenografted cells is enhanced by stable knockdown of Notch2 [88]. Notably, this increased* in vivo* tumor growth is determined by the increase in cytokines secretion and Notch1 activation, thus suggesting a compensatory response of cancer cell [88]. More interestingly, numerous studies suggested that Notch2 overexpression is related to a greater chance of survival of breast cancer patients [89]. Parr and colleagues analyzed Notch-1 and Notch-2 mRNA and protein expression levels in normal and breast cancer tissues also in association whit clinicopathological parameters [89]. The results showed that high level of either Notch1 mRNA or protein is associated with a poorer outcome for patients while a high expression of Notch2 is correlated with a better prognosis. In addition, the authors demonstrated an opposite expression of Notch1 and Notch2 proteins during tumor development, related to its differentiation state. Regarding Notch2 gene mutational pattern in TNBC samples, many focal amplifications were also found in its PEST or HD domain: in particular, the PEST domain showed six mutations, three of them leading to a gain of function, while the HD domain exhibited two missense mutations, finally leading to Notch2 overexpression [40].

All these data suggest that Notch-2 role remains ambiguous in TNBC. However, to date there is much more evidence to support the view that it should have a tumor suppressive role rather than an oncogenic role.

#### 2.2.3. Notch3

As we have previously described, TNBCs are genetically unstable and they are usually characterized by a complex pattern of genetic aberrations such as focal amplifications. On the basis of the evidence that Notch3 is highly expressed in TNBCs [51], Turner and colleagues subjected a wide subset of TNBCs to high resolution microarray-based comparative genomic hybridization and to genome-wide gene expression analysis in order to model mutational signatures of Notch3 gene. The obtained results highlighted that Notch3 gene amplification is quite recurrent and it is significantly overexpressed when amplified [90]. Furthermore, a broad spectrum of activating mutations that disrupt both HD and PEST domains, thus favoring N3ICD expression, were discovered in Notch3 gene [40].

In keeping with these findings, the presence of activating mutations, coincident with gene amplification and overexpression, lends genetic weight to the idea that there is a selective pressure to increase Notch3 activity for TNBCs initiation and progression. Indeed, the correlation between Notch3 signaling and TNBCs is corroborated by several studies.

First of all, it is already ascertained that Notch3 has transforming potentials* in vivo*, since transgenic mice overexpressing the intracellular domain of Notch3 (N3ICD) developed breast cancer [91]. In addition, Notch3 pivotal role in the proliferation of ErbB2-negative breast cancer cell lines has been demonstrated [92].

More recently, it was shown that Notch3 altered expression activates an oncogenic program in a panel of TNBCs. Selective Notch3 inhibition impairs tumor growth, whereas Notch3 agonism correlates with a malignant phenotype and increased proliferation. Indeed, transcriptomic analyses showed a Notch signature that includes overexpression of the c-Myc oncogene [93].

As occurred for cancers in general, TNBC malignancy correlates with tumor angiogenesis [94–96]. Reedijk and colleagues pointed out that Jagged1 and Notch3 are overexpressed in blood vessels of primary breast cancer [97], but little is done to understand whether Jagged1 and Notch3 are closely related to angiogenesis in TNBCs. Recently, Xue and colleagues speculated on the possible crosstalk between VEGF and Notch signaling in TNBCs [98] but further studies are needed. In addition, they showed that Jagged1 and Notch3 are detected in TNBCs at significantly higher levels than in no-TNBCs and their expression leads to more aggressive clinicopathological characteristics and poorer prognosis, confirming previous studies [56]. Moreover, RNAi-mediated depletion of Jagged1 and Jagged2 proteins in ErbB2-negative breast cancer cell lines inhibited proliferation and induced apoptosis* in vitro*, thus demonstrating an important autocrine/juxtacrine loop between Jagged1/Jagged2 ligands and Notch3 in TNBC context [99], which was then also observed in other tumor contexts [100].

In general, in about 50% of breast cancer patients bone is recognized as the first site of metastasis and TGF*β* plays a central role in this process [101]. Increasing evidence suggested that cancer cells interact with the bone microenvironment in order to promote the initiation and progression of bone metastasis [102]. Zhang and colleagues focused their attention on Notch3 and bone metastasis potential relationship in TNBCs: they observed that both osteoblasts and their secretion of TGF*β* increased Notch3 expression in TNBC cells that reside in the bone marrow niche. Notably, the inhibition of Notch3 expression is able to reduce osteolytic bone metastasis in xenograft animal models of TNBCs [103].

All these data supported the hypothesis of Notch3 involvement in promoting TNBC invasiveness and cancer cell seeding to secondary organs, thus being able to influence the acquirement of the metastatic phenotype and to complete the invasion-metastasis cascade. In this view, Leontovich and colleagues demonstrated that the MDA-MB-231 LM cells, isolated from experimental lung metastasis (LM), showed higher self-renewal capacity with respect to parental cells thanks to the upregulation of Notch3 reprogramming network.* In vitro* inhibition of Notch3 impaired the invasive capacity of MDA-MB-231 LM cells and interfered with late stages of the invasion-metastasis cascade. Interestingly, the pivotal role of Notch3 in determining an invasive phenotype and worst outcome was corroborated in unique TNBC cells resulting from a patient-derived brain metastasis [104].

Recently, some studies reported different molecular mechanism by which Notch3 seems to inhibit EMT in breast cancer [105, 106], including TNBCs [107], but overall high transcript levels of Notch3 were associated with less distant metastasis and better prognosis only in ER+ breast cancer [105, 106, 108].

Currently, several groups focus on the understanding of how the tumor microenvironment dictates treatment response. For instance, stromal cells sustain cancer cell survival after genotoxic and targeted therapy through paracrine and juxtacrine signaling [109]. In particular, it was demonstrated that stromal cells expressing Jagged1 on their surface were able to activate Notch3 on TNBC cells, thus promoting the expansion of cells resistant to chemotherapy and reinitiating tumor growth [110]. Therefore, these data supported the Notch3 role in chemoresistance of TNBCs.

Furthermore, Notch3 seems to be also involved in the resistance to targeted treatments, such as tyrosine kinase inhibitors (TKIs) against EGFR [111]. Targeting EGFR may be a promising approach to treat TNBCs since it is commonly overexpressed in this breast cancer subtype [112], but several clinical trials failed due to intrinsic and acquired resistance. In this scenario, the authors demonstrated a novel role of Notch3 in promoting resistance to TKI-gefitinib through regulating EGFR localization, thus rendering it targetable by TKI-gefitinib [111].

Overall, these studies suggested that Notch3 is strictly associated with pathogenesis of TNBCs and it is responsible for their aggressive phenotype.

#### 2.2.4. Notch4

The first evidence that Notch4 could function as a protooncogene was associated with mouse mammary tumors which showed integration of the mouse mammary tumors virus (MMTV) into the Notch4 locus [113]. The major consequence of this integration is the production of a truncated protein which is constitutively activated. Therefore, aberrant expression of Notch4 leads to mammary epithelial dysplasia and impaired differentiation, finally resulting in mammary tumorigenesis in mice [114].

Several studies documented a correlation between TNBCs and high expression of Notch4. Speiser and colleagues analyzed 29 TNBC-bearing patients and Notch4 was widely expressed in 73% of the cases [53], in agreement with a previous study [115]. Moreover, Wang and colleagues analyzed a wider panel of breast cancers (98 samples) in which TNBCs exhibited the highest Notch4 expression [116], thus suggesting a pivotal role of Notch4 receptor in this subtype. This was further confirmed from genome-wide analysis of TNBC human samples in which Notch4 was found commonly mutated in patients with progression free survival (PFS) less than 3 months [41]. Notch4 seems to be associated with metastatic TNBCs: Lawson and colleagues, by analyzing the transcriptomic signature of TBNC patient-derived xenografts, detected high levels of Notch4 in metastatic cells [117]. In accordance with these findings, the expression of Notch4 correlated with overall poor prognosis and experimental evidence indicates that Notch4 contributed to tumor invasion and metastasis by sustaining EMT at the invasive front of primary tumors [118]. Castro and colleagues performed* in vivo* experiments on mice that established spontaneous lung metastasis from JygMC(A) cells. The authors state that Notch4 promoted tumor growth and metastasis through the finding of Notch4 nuclear localization in both primary tumors and lung metastasis. The treatment with an orally active GSI inhibitor (RO4929097) reverted the phenotype, thus inhibiting primary tumor growth, reducing the number of metastatic lung nodules, and finally confirming the contribution of Notch4 during mammary tumor progression [118]. More recently, Castro and colleagues tested Sulforaphane (SFN) in both human and murine TNBC cells and they observed that the same JygMC(A) cells were more resistant to SFN. Molecularly, the authors demonstrated that SNF is able to reduce the promoter activity of Cripto1, a known positive regulator of Notch receptor maturation and signaling [119], thus linking the Cripto-mediated Notch4 signaling impairment with the observed inhibition of the proliferation of breast CSCs [120]. As previously mentioned, CSCs are associated with high-grade breast cancer and distant metastasis [121, 122] and contribute to intratumor heterogeneity [123]. Therefore, the understanding of signaling networks that regulate CSCs is urgently required. Since stem cells and cancer stem cells are usually characterized by the activation of the same pathways and Notch4 has been implicated in mammary stem cells [124], during the last decade several studies demonstrated that Notch4 activity strongly correlated with self-renewal and chemoresistance of breast cancer stem cells (BCSCs). Harrison and colleagues isolated BCSCs from breast cancer cell lines and primary breast cancer samples. They compared the activation of Notch1 and Notch4 in BCSC-enriched population to differentiated cells and they found that Notch1 and Notch4 are differentially expressed: Notch1 promotes the proliferation of progenitor cells and sustains their differentiation whereas Notch4 plays a role in the commitment of BCSCs to progenitor cells. Interestingly, decreased levels of Notch4 (but not of Notch1), obtained by both RNA interference or pharmacological treatment, significantly reduced mammosphere formation* in vitro* and reduced tumor formation* in vivo*, thus suggesting a specific role of Notch4 in regulating this subpopulation [125]. These results were consistent with a previous study in which Notch4-neutralizing antibody is able to inhibit cancer stem cell activity* in vitro* [126].

In keeping with these data, Rustighi and colleagues found that Notch1/4 is involved in the maintenance of breast stem cell self-renewal. The authors pinpointed the role of the prolyl-isomerase Pin1 in sustaining high levels and transcriptional activity of Notch1/4 through preventing their E3-ligase FBXW7-dependent proteasomal degradation [127, 128]. More interestingly, the authors demonstrated that the Notch1/4 suppression, Pin1-dependent, correlated with a sensitization of BCSCs to chemotherapy* in vitro* and* in vivo* [128].

All together these results suggest that high Notch4 levels are crucial to promote mesenchymal signature and to keep pro-stemness signaling constant during tumor progression of TNBC.

## 3. Notch-Targeting Approaches and Clinical Perspectives in TNBC

Chemotherapy is the current primary therapy for TNBCs in the neoadjuvant, adjuvant, and metastatic settings [129]. Although there is a small subgroup of patients with TNBC for whom chemotherapy may be effective, the heterogeneity of these tumors requires the development of most promising new targets and associated therapies that may improve the outcome of TNBC-bearing patients. The deregulation of various signaling pathways has been confirmed in patients suffering from TNBC and has recently come under development as a novel treatment option [130]. Among them, ADP ribose polymerase (PARP) inhibitors named PARPi (olaparib, veliparib, rucaparib, niraparib, talazoparib, and CEP-9722) have been evaluated on TNBC patients as mono- or combination therapies. Interestingly, BRCA mutated tumor cells are more sensitive to PARPi for combined loss of PARP and homolog recombination repair [131]. Tyrosine kinase receptors targeted by therapy include epidermal growth factor receptor (EGFR), fibroblast growth factor receptor (FGFR), and vascular endothelial growth factor receptor (VEGFR) [90]. Expression of EGFR has been reported in up to 89% of TNBC patients, particularly for BL2-subtype tumors [132], which depend on EGFR for proliferation and represent the major candidates for anti-EGFR therapies [133]. Unfortunately, only limited benefit has been reported in clinical trials using anti-EGFR agents, such as monoclonal antibodies (Cetuximab or Panitumumab), in combination with chemotherapy [134, 135]. Defect of Wnt/*β*-catenin pathway has been identified as an alternative therapeutic approach [136] and PI3K/AKT/mTOR pathway is also emerging as a promising target. It has been reported that inhibition of the PI3K pathway enhanced sensitivity to PARPi in TNBC cell lines [137]. Moreover, Yunokawa et al. reported positive effects of Everolimus, an mTOR inhibitor [138]. For years, TNBC was not considered sensitive to immunotherapy, but now this option is emerging as an exciting treatment [139], because of the immunogenic nature of TNBC compared with other breast cancer subtypes [140]. However, these strategies are effective in less than 20% of cancer patients or are useful only for certain TN cancer subgroups [141]. Therefore, further therapeutic strategies are urgently needed.

In this scenario, targeted therapy focused on modulating aberrant Notch signaling is emerging as a possible treatment approach for patients with TNBC (Table 1). Novel opportunities arise from the discovery of Notch crosstalk with many oncogenic signaling which suggested that Notch pathway may be considered such a multitarget drugs' candidate [13, 142–144]. To date, several clinical studies involved targeting of Notch pathway with either *γ*-secretase inhibitors (GSIs) or monoclonal antibodies (mAbs) against Notch receptors [145], which represent the major therapeutic targets of Notch signaling pathway.

### 3.1. *γ*-Secretase Inhibitors (GSIs) in TNBC

GSIs act by preventing the cleavage of the active form of all Notch receptors, thus inhibiting their transcriptional activity [146, 147]. It is demonstrated that GSIs interfere with cell cycle, lead to apoptosis in both luminal and TNBC cell lines [14], and, in particular, reduce the growth and dissemination of MDA-MB-231 TNBC xenografts [148]. It is shown that GSI treatment upregulates the proapoptotic protein Phorbol-12-myristate-13-acetate-induced protein 1 (NOXA), reduces CSC colony formation, and results in apoptosis of human TNBC cell lines [149]. In another study, it is demonstrated that 13% of TNBCs with PEST domain mutations in NOTCH1, NOTCH2, and NOTCH3 receptors and patient-derived xenografts are highly sensitive to the PF-03084014 GSI [40]. These mutations provoke a truncation in the C-terminus of Notch protein, removing the PEST domain while retaining the *γ*-secretase cleavage site. These findings suggest that GSI might be promising in treatments of TNBC subset with specific Notch sequence alterations.

Unfortunately, the gastrointestinal negative effects impede the clinical use of GSIs [150], suggesting that much more work is required for having favorable effects after GSI treatments. In this scenario, novel therapeutic strategies will likely come from combinations of GSIs with conventional chemotherapy, in order to reduce the single dose of both treatments, thus limiting either toxicity. Zhi-Lu Li and colleagues demonstrated the feasibility of the combined use of GSIs and Doxorubicin on MDA-MB-231 cells, resulting in encouraging new therapeutic approach in TNBC treatment [74]. Actually, RO-4929097 and MK0752 GSIs are investigated in phase I/II clinical trials and, recently, the combination of RO-4929097 and chemotherapics like Paclitaxel and Carboplatin is in a phase I clinical trial for TNBCs [151]. Moreover, since preclinical studies prompted evaluation of combination of PF-03084014 GSI with docetaxel for the treatment of patients with TNBC [152, 153], Locatelli and colleagues designed a phase I study in order to evaluate safety, tolerability, pharmacokinetics, and antitumor activity of this combination. Preliminary results demonstrated feasibility of the combined GSI-chemotherapy approach, thus promoting further studies in order to use Notch signaling inhibitors in combination with conventional chemotherapy in the treatment of TNBC-bearing patients [154].

### 3.2. Monoclonal Antibodies (mAbs) in TNBC

Despite these encouraging results on GSI treatment, there is an increasing number of studies based on the use of monoclonal antibodies against Notch members in order to achieve higher specificity. The use of specific monoclonal antibodies is based on their capacity to bind the extracellular regulatory region of the receptor, to mask the cleavage domain of metalloproteinase ADAM, and to induce a conformational change of the receptor into its inactive form [155]. Recently, it has been shown that an antibody against the negative regulatory region (NRR) of Notch1 resulted in reduced proliferation, restricted expression of its targets HES1, HES5, and HEY-L, reduced colony forming ability, and lessened cancer stem-like population in MDA-MB-231 cell lines [156]. As previously mentioned, the inhibition of Notch1 with the novel monoclonal antibody MAb602.101 reduced TNBC cell lines tumor growth and sphere-forming potential, thus directly affecting CSCs niche [66]. In accordance with these results, TNBC patients which display high level of Notch1 expression are characterized by poorer survival, thus suggesting that hyperactivation of Notch1 receptor may be used as a predictive marker for TNBCs [66] and finally pointing out the Notch1 inhibition as a potential novel approach to achieve the outcome of TNBC-bearing patients. Interestingly, it has been also demonstrated that the antibody use can amplify chemotherapy treatments: in a TNBC patient-derived xenograft model, Notch1 monoclonal antibodies exhibited synthetically antitumor efficacy combined with docetaxel via inhibition of CSCs generation and maintenance [64].

Moreover, a Notch2/3 blocking monoclonal antibody named tarextumab (OMP-59R5) was developed: it was successfully tested on patient-derived epithelial tumor xenograft models, including breast, thus showing significant antitumor activity [157]. Recently, Choy and colleagues used a novel monoclonal antibody that selectively targets the Notch3 NRR (anti-N3.A4) [158] to make a comparison between Notch3-specific versus pan-Notch effects for treatment of TNBCs. They documented that both treatments significantly inhibited colony formation* in vitro* and modestly reduced tumor growth* in vivo* to similar extent [93]. Therefore, the authors strongly suggested that the therapeutic targeting of Notch3 could provide therapeutic benefit without the known toxicities associated with pan-Notch inhibition, as GSIs fail to distinguish the particular Notch receptor driving growth [93]. Similar results have been obtained by Farnie and colleagues who demonstrated that Notch4-neutralizing antibody inhibited cancer stem cell activity* in vitro* [126].

Notch ligands targeting could be also a promising strategy to reduce Notch activation. Hoey and colleagues used monoclonal antibody against DLL4 ligand to block its binding to Notch1, thus observing antitumor effects in a wide range of human tumor xenografts from various tumor types, including breast cancer. Specifically, the inhibition of DLL4-Notch1 axis decreased CSC frequency [159]. More recently, a monoclonal antibody against Jagged1 ligand has been developed to be used for the treatment of established bone metastasis that is refractory to chemotherapy [160]. The authors observed that chemotherapy agents were able to induce Jagged1 expression at the cell membrane of osteoblasts and mesenchymal stem cells of bone marrow, which in turn activated Notch signaling, finally promoting chemoresistance [160].

Interestingly, more recently it has been demonstrated that the overexpression of Notch receptors or their ligands at the cell membrane of cancer cells might be also turned to our advantage in order to effectively deliver cytotoxic agents to the tumor sites. In this view, a novel anti-Notch3 antibody-drug conjugate currently named PF-066580808 is now under clinical investigation (phase I) for the treatment of breast cancer, including TNBCs [161]. Besides above described approaches, several natural compounds and their derivatives are showing Notch inhibition and antiproliferative activities in different* in vitro* cancer models, thus suggesting their potential application as additional therapeutic option in Notch-related cancers [68, 162].

Further studies into mechanisms of action of individual Notch receptor in TNBC development and behavior should be addressed in order to ameliorate the understanding of the complexity and mechanisms that underlie TNBCs. In this view, the aforementioned results suggest that the potential targeting of the Notch signaling pathway with different molecules should be studied in more detail to further improve the treatment options for TNBC-bearing patients.

## 4. Conclusion

TNBC is an aggressive subgroup of human breast cancer, characterized by high rates of relapse and frequent metastasis. Since unresponsiveness to current treatment is often observed, the development of novel strategies to treat also this form of breast cancer is urgently required.

Several pathways are involved in the pathogenesis of TNBC. Among them, Notch signaling plays a key role in tumor initiation and mainly in tumor progression. Indeed, several experimental studies documented the role of Notch signaling in promoting EMT for cancer cell seeding to secondary organs and in sustaining the maintenance of CSCs which are responsible for chemoresistance. Therefore, inhibition of Notch signaling has been considered as an attractive strategy for the treatment of TNBC. Several pan-Notch inhibitors are currently under clinical trials in combination with chemotherapy [163] but they fail to distinguish individual Notch receptors and cause intestinal toxicity. In addition, since individual Notch receptors can have opposite role in the same cancer, their simultaneous inhibition may have pleiotropic effects possibly resulting in tumor stimulation.

**Figure 2:**
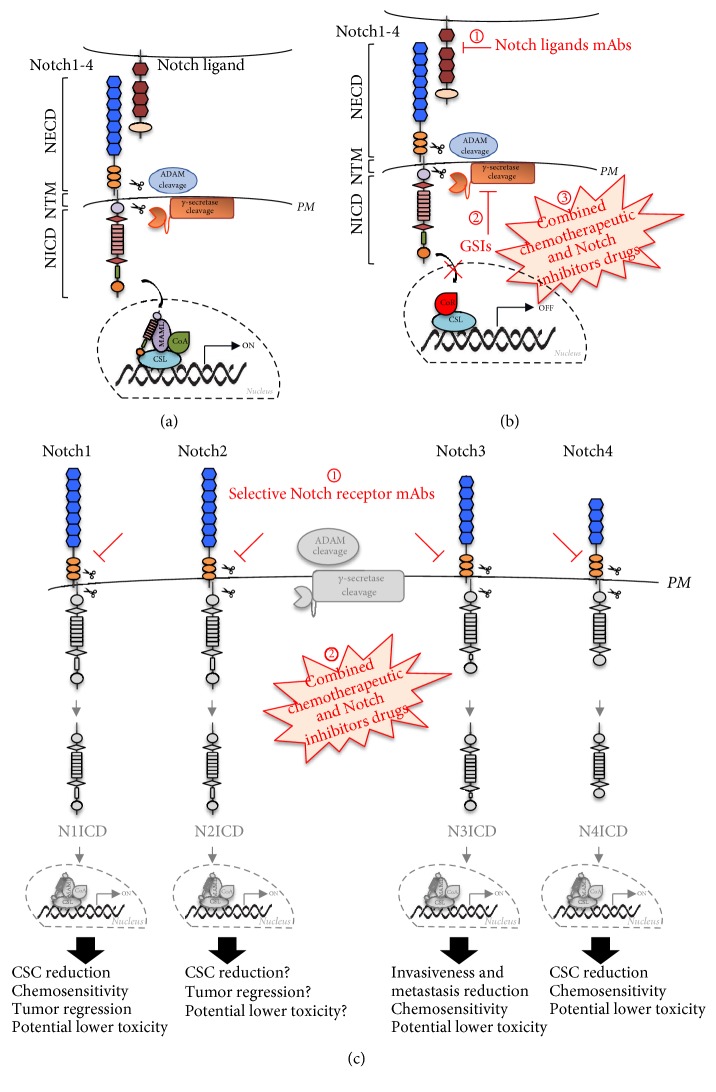
*Notch-targeting therapeutic approaches in TNBCs.* (a) The canonical Notch signaling pathway: ligand binding promotes sequential cleavages of the Notch receptors (Notch1-4) by ADAM enzyme and *γ*-secretase complex, resulting in the release of NICD, which translocates in the nucleus, interacts with transcriptional regulators to transcriptionally activate the canonical Notch target genes (ON), thus leading to the regulation of TNBC growth and progression. (b) Notch inhibitors with lower or absent selectivity, respectively, include mAbs targeting the Notch ligands and GSIs. (1) mAbs against Notch ligands prevent ligand-receptor interaction and the subsequent Notch cleavages, preventing Notch signaling triggering. Little is known about the specific Notch-ligand relationship in TNBC; thus further studies are needed to consider ligand blocking as a potential alternative selective approach in TNBC treatment. (2) GSIs act as pan-Notch inhibitors since they prevent the cleavage of all Notch receptors, thus avoiding the release of any NICD. This unselective mechanism of action is strongly correlated with a high intestinal toxicity in patients, which significantly impairs their clinical use. (3) Lower doses of GSIs used in combination with chemotherapeutic drugs result in improved clinical outcome and less toxicity, which however must be overcome. (c) A higher selectivity can be obtained by using monoclonal antibodies directed against the extracellular domain of a specific Notch receptor (1): mAbs mask the cleavage domain of ADAM, thus preventing the binding of this enzyme and the subsequent *γ*-secretase cleavage. The final effect will depend on the specific block of the single Notch receptor, also used in combination with chemotherapeutic drugs (2). Several studies detailed in the text have suggested that a greater selectivity in the Notch inhibition approach for TNBCs treatment is strongly correlated with a higher probability of success in favoring tumor regression, associated with less toxicity and therefore with a potential better prognosis of TNBC-bearing patients. Abbreviations. ADAM: a disintegrin and metalloproteinase; CSL: CBF1/Su(H)/Lag-1; CoA: coactivator; CoR: corepressor; GSIs: *γ*-secretase inhibitors; mAb: monoclonal antibody; MAML1: mastermind-like 1; NECD: Notch extracellular domain; NICD: Notch intracellular domain; NTM: Notch transmembrane;* PM*: plasmatic membrane.

This review covers the roles of individual Notch receptors in TNBC development and progression, thus showing that they only partly share the same functions in TNBC context. As a result, determining the Notch receptor which is specifically involved in different TNBC subtypes might be useful to identify patients who are most likely able to respond to different targeted therapy, paving the way for avoidance or likely reduction of the therapeutic complications associated with nonselective Notch inhibitors. In conclusion, this review will aid further research in identifying a suitable treatment for TNBC, as the specific inhibition of a single Notch receptor or ligand might promote new clinical trials aiming to evaluate more selective and less toxic alternatives for Notch inhibition in the treatment of TNBC-bearing patients (Figure 2).

## Figures and Tables

**Table 1:** Summary of notch receptors-related processes and treatments in TNBC.

Notch receptor	Process	Refs	Treatment	Studies	Refs
Notch1	Tumor growth	[59–61]			
	Mitochondrial metabolism	[60, 80]	mAbs	Preclinical	[156]
	Regulation of cancer stem cells	[64, 65, 68]	mAbs (+chemotherapeutic agents)	Preclinical	[64, 66, 156, 159]
	Drug resistance	[67–70, 73, 75]	GSI + chemotherapeutic agents	Preclinical and clinical	[74, 151–153]
	Invasion and metastasis	[59, 71, 73]			

Notch2	Tumor growth	[87, 88]			
	Regulation of cancer stem cells	[84, 85]	mAbs	Preclinical	[157]
	Invasion and metastasis	[84, 85]			

Notch3	Tumor growth	[92, 93]			
	Angiogenesis	[97, 98]	mAbs	Preclinical and clinical	[93, 157, 161]
	Drug resistance	[110, 111]			
	Invasion and metastasis	[103, 104, 107]			

Notch4	Regulation of cancer stem cells	[125–128]	mAbs	Preclinical	[126]
	Invasion and metastasis	[117, 118]	GSI	Preclinical	[118]

Abbreviations. mAbs: monoclonal antibodies; GSI: *γ*-secretase inhibitor.
